# Immunological predictors of overall survival in treatment naïve metastatic pancreatic cancer patients

**DOI:** 10.1186/2051-1426-3-S2-P85

**Published:** 2015-11-04

**Authors:** Matthew R Farren, Thomas Mace, Susan Geyer, Sameh Mikhail, Christina Wu, Kristen Ciombor, Sanaa Tahiri, Daniel Ahn, Anne Noonan, Miguel Villalona-Calero, Tanios Bekaii-Saab, Gregory Lesinski

**Affiliations:** 1 Department of Internal Medicine, James Comprehensive Cancer Center, The Ohio State University, Columbus, OH, USA; 2 Health Informatics Institute, University of South Florida, Tampa, FL, USA

## Background

Pancreatic ductal adenocarcinoma (PDAC) is an aggressive cancer that has a 5-year survival rate of less than 7% and is ultimately refractory to most treatments. An assessment of immunologic factors relevant to disease has not been performed in a comprehensive manner for treatment naïve patients to date. We hypothesized that systemic immunologic activation and/or immunosuppression would predict overall survival (OS) in treatment naïve PDAC patients.

## Methods

To test this hypothesis, peripheral blood was collected from 73 patients presenting with previously untreated metastatic PDAC who went on to be treated in a randomized multi-center Phase II trial (NCT01280058: Carboplatin and paclitaxel with or without viral therapy in treating patients with recurrent or metastatic pancreatic cancer).

## Results

There was no significant difference in outcomes between treatment arms in the subsequent clinical trial, and across both arms OS was 7.5 months. Extensive immunologic profiling was conducted to assess relationships between overall survival and the level of soluble plasma biomarkers (cytokines, chemokines and growth factors) or detailed immune cell phenotypes as measured by flow cytometry at baseline, prior to any treatment. Higher levels IL-6 and IL-10, prominent immunosuppressive cytokines were negatively associated with OS (p = 0.008 and 0.020, respectively; HR = 1.157 and 1.277, respectively). Conversely, higher levels of the monocyte chemoattractant MCP-1 were associated with significantly longer OS (p = 0.045; HR = 0.687). Patients with a greater proportion of antigen-experienced T cells (CD45RO^+^) had longer overall survival (CD4 p-value = 0.032; CD8 p-value = 0.036; HR = 0.357 and 0.614, respectively). While greater expression of the T cell checkpoint molecule CTLA-4 on CD8^+^ T cells was associated with significantly shorter overall survival (p = 0.0198; HR = 1.533), the TIM3 molecule had a positive association with survival when expressed on CD4^+^ T cells (p = 0.0269; HR = 0.622). Overall, these data identify several immunological predictors of overall survival in a treatment naïve cohort of patients with metastatic pancreatic ductal adenocarcinoma.

## Conclusions

This supports the idea that therapies targeting or modifying the immune system could improve future PDAC treatment outcomes. To our knowledge, this work represents the largest cohort and most comprehensive immune profiling of treatment-naïve metastatic PDAC patients to date.

